# Acorn worm ossicle ultrastructure and composition and the origin of the echinoderm skeleton

**DOI:** 10.1098/rsos.220773

**Published:** 2022-09-21

**Authors:** Charles Larouche-Bilodeau, Christopher B. Cameron

**Affiliations:** Département de sciences biologiques, University of Montreal, Montreal, Quebec, Canada H3C 3J7

**Keywords:** biomineralization, Ambulacraria, acorn worm, Enteropneusta, evolution, Deuterostomia

## Abstract

Here, we describe the shape and mineral composition of ossicles from eight acorn worm species, bringing the total known biomineralizing enteropneusts to 10 and confirming that ossicles are widespread in Enteropneusta. Three general forms were identified including a globular form that occurs in all three major enteropneust families. The biomineral compositions included all three polymorphs of calcium carbonate; calcite, aragonite and vaterite, and low to high magnesium concentrations. Calcite was the most common and characteristic of echinoderm ossicles. Based on these findings we hypothesize that an enteropneust-like ancestor to the Ambulacraria had ectodermal ossicles, formed in an extracellular occluded space bordered by a sheath of sclerocyte cells. The ossicles were microscopic, monotypic globular shaped, calcite ossicles with low to high Mg content and MSP130 proteins. The ossicles lacked intercalation with other ossicles. The function of acorn worm ossicles is unknown, but the position of ossicles in the trunk epithelia and near to the surface suggests predator deterrence, to provide grip on the walls of a burrow or tube, as storage of metabolic waste, or to regulate blood pH, rather than as an endoskeleton function seen in fossil and crown group Echinodermata.

## 1. Background

Biominerals in the animal kingdom range from the simple statoliths found in xenacoelomorphs, ctenophores and placozoans, to the extensive shells of molluscs, the reef-forming skeleton of sponges and corals, and the vertebrate skeleton [1–5]. Most biomineralizing phyla use a form of calcium crystals including calcium carbonate or calcium phosphate [6]. In animals, mineralized tissue appeared during the Ediacaran-Cambrian transition, most likely via convergent or parallel evolution, though the number of independent origins is unclear [6–9]. Among the deuterostome animals, the vertebrates and the echinoderms have extensive biomineralized tissues. That of echinoderms is composed of biomineralized ossicles that intercalate to form an extensive skeleton, except in Holothuroidea [10]. It is formed of many ossicles that grow in extracellular occluded spaces surrounded by a sheath of sclerocyte cells. Each ossicle acts optically like a single crystal [11]. The ossicles are composed of high magnesium calcite, a polymorph of calcium carbonate, commonly organized into a porous microstructure called stereom [12]. In echinoderms, biomineralization involves many proteins like the SM30, SM50 and MSP130 protein families, metalloproteases and carbonic anhydrases [13,14]. MSP130 proteins are occluded directly in the calcite ossicles.

Echinoderms with hemichordates form the deuterostome clade Ambulacraria. Hemichordata is a small phylum of exclusively marine animals that can be found at all latitudes and depths comprising two major classes: the tubicolous colonial pterobranchs and the benthic vermiform enteropneusts. Pterobranchs lack biomineralized tissue but acorn worms form biomineralized microscopic ossicles [15]. The class Enteropneusta is composed of four families that are differentiated based on soft body morphological characteristics [16–20]. The two enteropneusts currently known to form biomineralized ossicles are members of distantly related families: the direct-developing harrimaniid *Saccoglossus bromophenolosus* and the indirect developing ptychoderid *Ptychodera flava,* which suggests that ossicles may be widespread among Enteropneusta [9,15] and perhaps an ancient feature of Ambulacraria [15].

Hemichordate ossicles are ectodermal. They form in an extracellular occluded space formed by the sheath of sclerocyte cells. The ossicles of *Saccoglossus bromophenolosus* are composed of aragonite, rather than the echinoderm calcite, and they have a low concentration of magnesium [15]. The ossicles have a mesh-like microstructure on the surfaces and are solid internally [15]. The *Saccoglossus kowalevskii* genome includes genes homologous to the echinoderm Sp-Msp130 L, Sp-Msp130, Sp-Msp130r1, Sp-Msp130r2, Sp-Msp130r3, Sp-Mt1-4/MmpL7 (matrix metalloproteases) and Sp-Clara7LA (carbonic anhydrases) (Glean3_entries 06 387; 02 088; 13 822; 16 506; 13 823, 28 748, 12 518, respectively). There is no direct proteomic sequence data from acorn worm ossicles, but these data provide tantalizing evidence that hemichordates and echinoderms ossicles may be homologous structures, although we need deeper knowledge of hemichordate ossicles before homology can be inferred or rejected [15,21,22].

**Figure 1.**
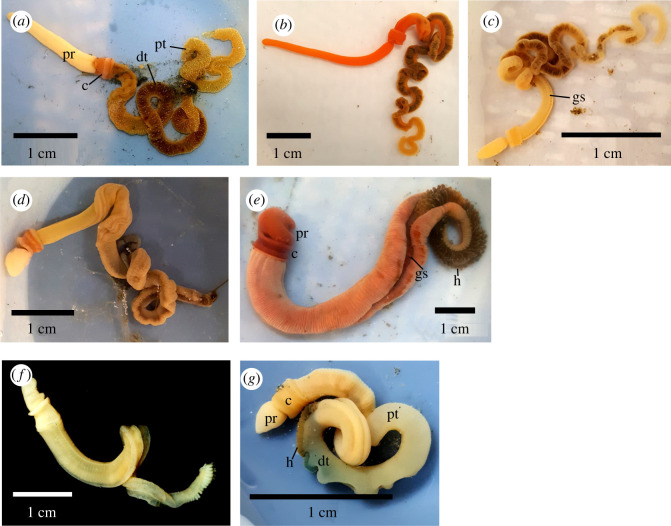
Photographs of acorn worms analysed in this study. (*a*) *Saccoglossus kowalevskii*. (*b*) *Saccoglossus pusillus*. (*c*) *Harrimania planktophilus*. (*d*) *Protoglossus graveolens*. (*e*) *Schizocardium californicum*. (*f*) *Glossobalanus berkeleyi*. (*g*) *Balanoglossus occidentalis*. (c, collar; dt, dark trunk; gs, gill slits; h, hepatic sacs; pt, pale trunk; pr, proboscis) (*a*,*b*,*c*,*d*,*e*,*g*) were photographed live, and (*f*) was fixed and stored in ethanol.

Here we described the ultrastructure and biomineral composition of ossicles from eight additional acorn worm species, from three families, quintupling the number of known biomineralizing acorn worms (figure 1). This study provides a broad sampling and characterization of enteropneusts ossicles, allowing a deeper understanding of the origin and evolution of enteropneust and echinoderm ossicles.

## 2. Material and methods

### 2.1. Acorn worm collection and fixation

Four acorn worm species from the family Harrimaniidae were collected. *Saccoglossus kowalevskii* ([23], figure 1*a*) were collected in Waquoit Bay National Research Reserve, Cape Cod, Massachusetts, from 1 to 30 September 2017, during the low tides. *Saccoglossus pussilus* ([23], figure 1*b*) and *Harrimania planktophilus* ([24], figure 1*c*) were collected in the intertidal zone of Cape Beale, British Columbia, Canada in May 2018*. Protoglossus graveolens* ([25], figure 1*d*) was collected in Lowe's Cove adjacent to the Darling Marine Center, Walpole, Maine, in May 2017 during the low tides*.* One species from the family Spengelidae, *Schizocardium californicum* ([26], figure 1*e*), was collected in Morro Bay State Park, Morro Bay, California, USA in June 2018, during the low tides. Three species of the family Ptychoderidae were collected. *Glossobalanus berkeleyi* ([19], figure 1*f*) on 5 May 2007, and *Balanoglossus occidentalis* (figure 1*g*) on 16 June 2018, were both collected in Penrose Point State Park, Washington, during the monthly low tides. *Balanoglossus aurantiacus* [23] were collected adjacent to the Duke University Marine Laboratory, North Carolina on 8 March 1996. Those species were chosen to provide a broad sampling of the enteropneust families and because they are easy to acquire. No Torquaratoridae were sampled because of their rarity and the destructive nature of our experiments.

All worms were transported from the collection site to a laboratory or incubator in bottles filled with seawater. Individual worms were then transferred to weigh boats that were submerged in flow-through seawater tables without sediment and allowed to vacate their guts for 12 h, at temperatures close to or colder than that of the seawater at the collection site. Once a worm gut was evacuated, it was cleaned using forceps, relaxed in a 7% MgCl_2_ seawater solution, and fixed in a 4% formalin solution in Borax-saturated water overnight. The solution was then changed to Borax saturated 70% ethanol solution for storage.

A culture of embryos from *S. kowalevskii* was started and kept for four months to describe when ossicles appeared and what they looked like in the first stages. The culture was provided by Christopher Lowe's laboratory from Waquoit Bay National Research Reserve, Cape Cod, Massachusetts on 22 September 2019. The embryos were cared for following [27].

### 2.2. Ossicle isolation and observation

To determine the presence of ossicles from different body regions of a worm, large worms were cut into longitudinal segments corresponding to different body regions and small worms (less than about 40 mm) were kept whole. Those regions were, from anterior to posterior, the proboscis, the collar, the pharyngeal region, post-pharyngeal gonadal region, the dark mid-trunk, the hepatic region (if it was present in the species) and the post-hepatic trunk. The epidermis of each sample was dissected from the gut and digested overnight in an Eppendorf tube with 7% NaClO (Javex bleach). If organic material was still visible, the bleach was replaced and the digestion step repeated. Once the soft tissue was digested, the bleach was replaced with distilled water, and the Eppendorf tube was left on a rack to allow the ossicles to settle.

To observe the ossicles, 50 µl of the ossicle precipitate was put on a glass slide with a coverslip and viewed with an Olympus BX51 compound light microscope with polarized filters and photographed with a Retiga 2000R camera using QCapture Pro 6.0 software. The samples were washed four times in distilled water and dehydrated through a graded series of ethanol. Changes were made by adding solutions to one side of the slide with a glass pipette while removing solution from the opposite edge using the capillary force of a Kimwipe. After two changes of 100% ethanol the coverslip was removed and the ossicles air dried. The areas of each slide with ossicles were then identified under a microscope and marked using a fine sharpie to facilitate observation under SEM. Using a diamond pen, the glass slides were trimmed and then glued to an SEM stub using double-sided tape and coated with gold. Electron micrographs were taken with either a Hitachi TM3030Plus environmental tabletop SEM, at the Integrated Quantitative Biology Initiative (IQBI), or a JEOL JSM-7600TFE field emission scanning electron microscope at Polytechnique Montreal Department of Mathematical and Industrial Engineering centre for characterization and microscopy of materials (CM)2. The latter provided the best resolution and had a lower secondary electron detector, the working distance was between 11.9 and 13.6 mm, the acceleration voltage was 5.0 kV, and the magnification was between 3 K and 25 K.

### 2.3. Ossicle biomineral composition

The mineral composition and crystalline polymorph of the calcium carbonate ossicles were determined with Raman confocal spectroscopy at University of Montreal department of chemistry Laboratoire de caractérisation des matériaux (LCM). Ossicles from the sea star *Pisaster ochraceus* and a valve fragment from the chiton *Katarina tunicata* were tested as controls for calcite and aragonite, respectively. Following the method of [15], the spectra were acquired using a Renishaw InVia Raman microspectrometer with a deep depletion CCD detector, 1800 l mm^–1^ grating and a holographic notch filter. Excitation was provided by a Spectra Physics argon ion 514.5 nm laser with 28 mW output and 5 mW at the sample. The spectrometer was mounted onto a Leica microscope with a 50× objective in an 1808 backscatter collection configuration. Using the formulae developed in [28], the concentration of magnesium (Mg) was calculated for each spectrum corresponding to calcite. The three CaCO_3_ polymorphs were then mapped onto a phylogenetic tree of the acorn worms using the echinoderm state calcite as the outgroup (figure 11) and optimized following parsimony.

## 3. Results

### 3.1. Ossicle shape and microstructure

**Figure 2.**
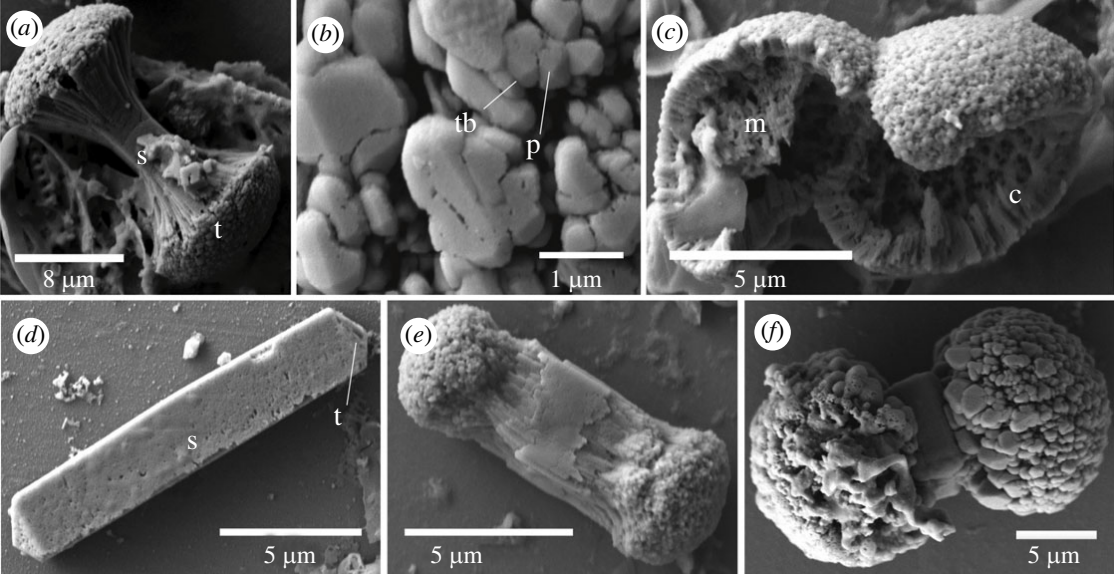
Scanning electron micrographs of ossicles from *Saccoglossus kowalevskii*. (*a*) Side view of a typical double-broccoli ossicle formed of a prismatic shaft (s) and two terminal lobes (t). (*b*) Close-up on a terminal lobe showing the arrangement and spacing of trabeculae (tb). The trabeculae end in a point (p). (*c*) Broken terminal lobes showing the brick-like arrangement of the cortex (c) and the porous medulla (m). (*d*) Side view of a typical prism ossicle formed of a prismatic shaft (s) and pyramidal tips (t). Most of the ossicle is the shaft. The surface of both the shaft and tip is porous. (*e*,*f*) Broccoli ossicle with small and large terminal lobes showing the variation in size.

Ossicles of the harrimaniid *Saccoglossus kowalevskii* (figure 1*a*) were found in the gonadal region of 23/50 worms and in the darkest mid-trunk of 45/50 worms. None we found from the proboscis, collar, or anterior pharyngeal trunk. *Saccoglossus kowalevskii* ossicles (figure 2) had a central shaft with two flared terminal lobes forming a doubled-ended broccoli shape. The shaft was smooth and square in cross-section (figure 2*a*). The terminal ends were made of single, columnal crystals that were near to square in cross-section, and pointed at the tip (figure 2*a,b*). The crystal surfaces did not show a mesh-like structure. Broken ossicles showed that these terminal crystals (length 1.3 ± 0.2 µm, *n* = 10) were arranged around a central porous lacuna (figure 2*c*). The terminal ends were absent (figure 2*d*), small (figure 2*e*), or sufficiently large that they almost touch medially, resulting in a medial groove (length = 20.6 ± 3.6 µm, *n* = 25, figure 2*f*). The lobed terminal ends widths correlated with the total ossicle length, with longer ossicles having larger terminal lobes (electronic supplementary material, figure S1, *R*^2^ = 0.6327, *n* = 25). A second type of ossicle was found that was a square prism shape with pyramidal ends (length = 14.5 ± 3.8 µm, *n* = 14, figure 2*d*). Pores with a maximum diameter of 1 µm were present on the surface (figure 2*d*). These may be an early developmental stage of the lobed form. Both types of ossicles were found in male and female worms, and in worms as short as 40 mm with immature gonads suggesting that biomineralization starts prior to sexual maturity. Ossicles were not found in juvenile worms up to the six gill-slit stage (the oldest worms that were cultured) suggesting that they first form at a later developmental stage.

**Figure 3.**
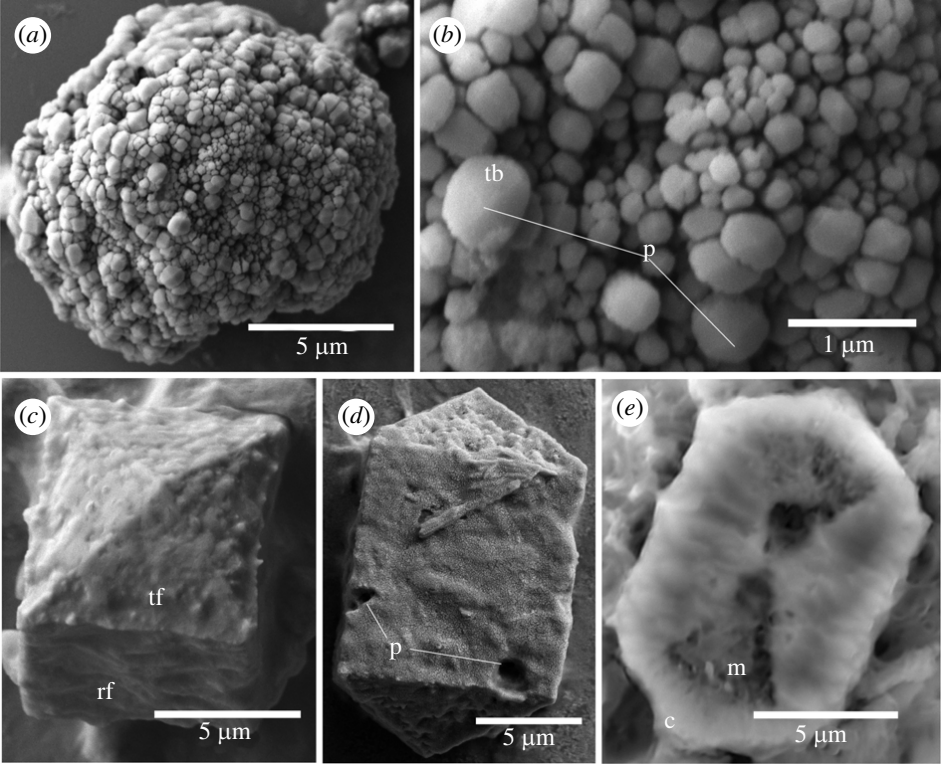
Scanning electron micrographs of ossicles from *Saccoglossus pusillus*. (*a*) Lone terminal lobe from a broccoli ossicle. (*b*) Close up on the same terminal lobe showing the arrangement and spacing of trabeculae (tb). The trabeculae end in a low point (p) and are rounded in cross-section. (*c*) Angled view of a typical prism ossicle. The triangular faces (tf) are rough, and the rectangular faces (rf) are wrinkly. This ossicle has no crack. (*d*) Prism ossicle with pores (p). Typical ossicle with a squared prism and pyramidal ends. (*e*) Prism ossicle with a bowtie-shaped crack. The crack shows a dense cortex (c) and a porous medulla (m).

Twenty-five *Saccoglossus pusillus* were subjected to dissection and epithelial digestion. Ossicles were only found in the pharyngeal and dark trunk regions of the worm body. Only six broccoli-shaped ossicles were found (figure 3), whereas prism-shaped ossicles, resembling ossicles without terminal lobe from *S. kowalevskii,* were common (figure 3*c*). All but one of the six broccoli-shaped ossicles were lost in the rinsing steps, and the one examined with SEM consisted of an isolated terminal lobe (figure 3*a*), so the shaft morphology is unknown. The terminal fragment was formed of an aggregate of polyhedral crystals resembling those of *S*. *kowalevskii*. Trabeculae of *S. pusillus* were columnar with an obtuse point at the tip and no visible mesh-like structure between the trabeculae in the same fashion as in *S. kowalevskii* (diameter = 0.4 ± 0.2 µm, *n* = 15). The trabeculae cross-sections were rounded (figure 3*a,b*) rather than angular like those of *S. kowalevskii* (figure 2*b*). The prism ossicles had transverse wrinkles on the rectangular faces and a rough texture on the triangular faces (figure 3*c*). Some ossicles had one or two isolated pores on their surface (diameter less than 1 µm; figure 3*d*). In a broken ossicle, a porous medula could be seen inside the cavity and a dense cortex at its margin (figure 3*e*). Ossicles ranged from 10 to 20 µm (14.6 ± 2.5 µm) in length and 6 to 11 µm (8.7 ± 1.4 µm) in width (*n* = 14).

**Figure 4.**
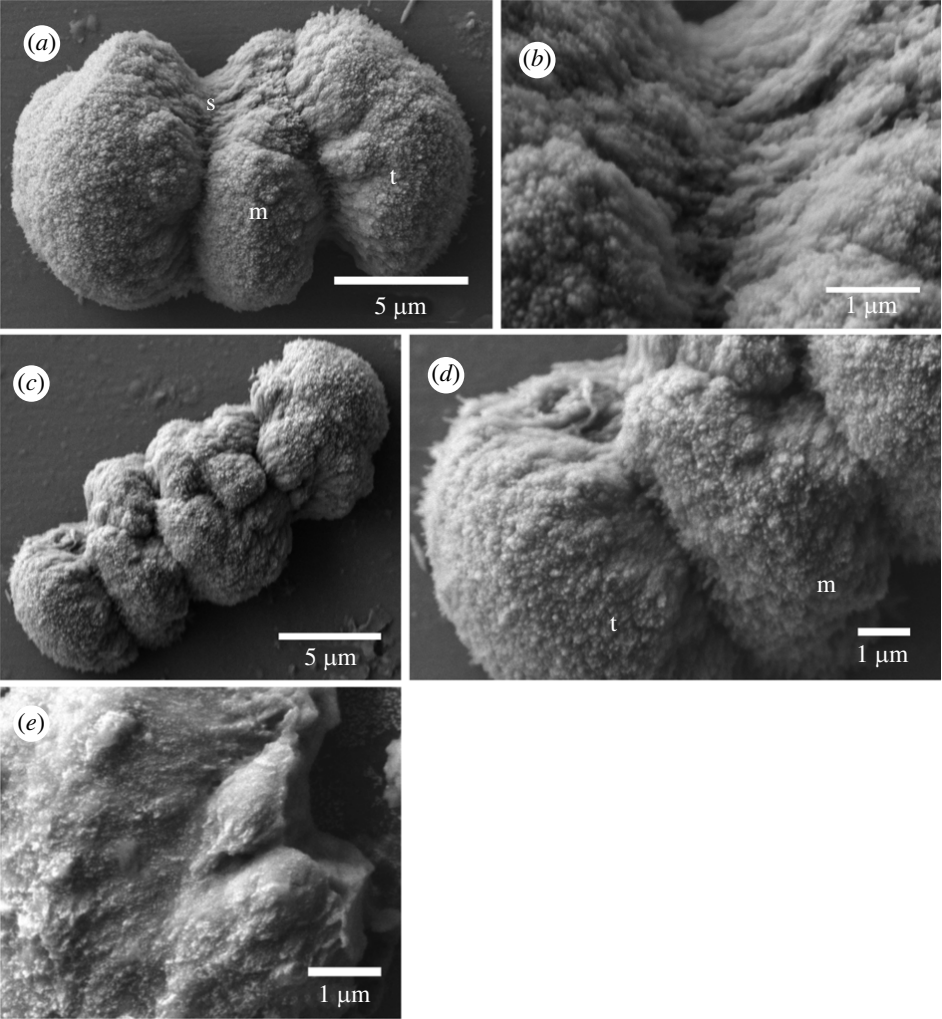
Scanning electron micrographs of ossicles from *Harrimania planktophilus*. (*a*) Sideview of a typical ossicle (m, medial lobe; s, shaft; t, terminal lobe). (*b*) Close-up on the shaft region of (*a*) showing its laminar organization. (*c*) The largest ossicle found, showing that the shaft is completely outgrown by medial lobes. (*d*) The structure of medial and terminal lobes are identical. Individual trabeculae are indistinguishable. (*e*) Broken terminal lobe of the ossicle shown in (*d*). There are no pores inside the ossicle.

The harrimaniid *Harrimania planktophilus* worm specimens were less than 40 mm in total length, so they were digested whole. Twelve ossicles were obtained from a whole animal, but the body region that bared the ossicles was not determined. The ossicles had a double-ended broccoli shape (figure 4), with the terminal lobes composed of trabeculae. These ossicles had a mean length of 12.4 ± 2.9 µm and a width of 9.1 ± 4.3 µm (*n* = 4). The lobe trabeculae were numerous, compact and too small to image individually, giving a coarsely textured surface (figure 4*b*)*.* Some of the ossicles had medial lobes between the terminal lobes (figure 4*c,d*). The grooves between lobes were formed of trabeculae that were continuous with trabeculae of the lobes (figure 4*b*). No shaft region was visible. The structure of the medial lobes was indistinguishable from the terminal lobes (figure 4*d*). The inside of the ossicle was solid, without pores (figure 4*e*).

**Figure 5.**
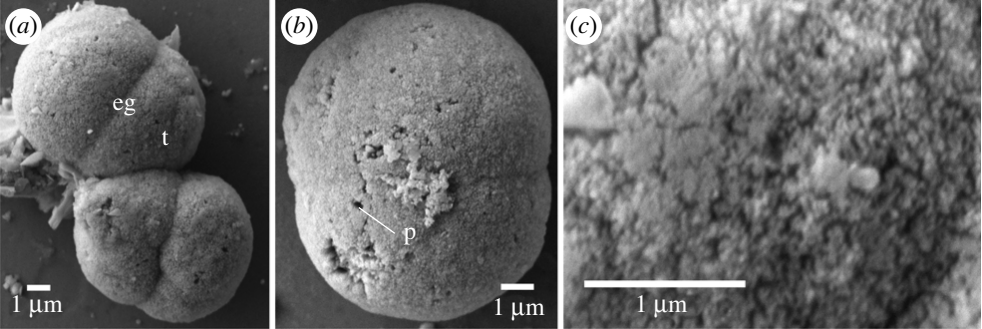
Scanning electron micrographs of ossicles from *Protoglossus graveolens*. (*a*) Two typical ossicles formed of two terminal lobes (t) separated by an equatorial groove (eg). (*b*) A larger ossicle with many pores (p). (*c*) The terminal lobe trabeculae are indistinguishable.

Ossicles of the harrimaniid *Protoglossus graveolens* were located throughout the length of the trunk and 40 ossicles were isolated from two worms, but most were lost in the rinsing steps. These ossicles had paired lobes but lacked a medial shaft (figure 5). These ossicles comprised two lobes with a distinct or a poorly developed medial groove between them (figure 5*a,b*). They had a mean length of 9.5 ± 2.8 µm and width of 7.7 ± 2.3 µm (*n* = 4). The lobes had a coursely textured surface with a few isolated surface pores (diameter less than 0.1 µm; figure 5*c*).

**Figure 6.**
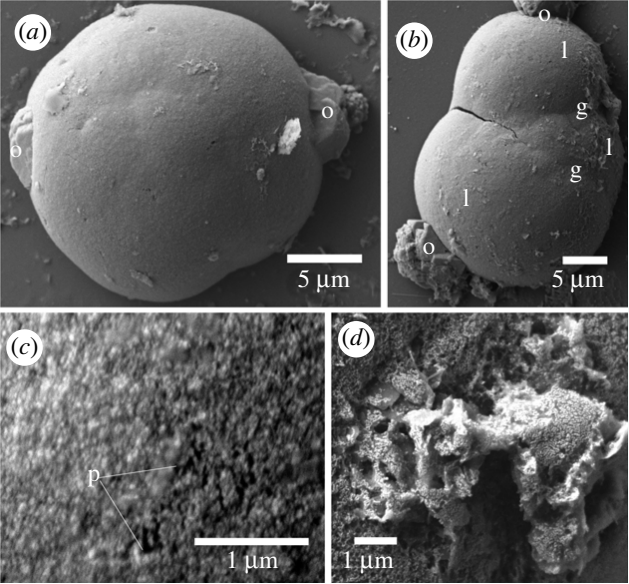
Scanning electron micrographs of ossicles from *Schizocardium californicum*. (*a*) An ossicle with a single lobe and terminal polyhedral outgrowths (o). (*b*) An ossicle with three lobes (l), separated by a groove (g). Polyhedral outgrowths are present. (*c*) Close-up on the surface of a lobe. The lobes may be an aggregate of smaller crystals and have pores (p). (*d*) Close-up of a broken polyhedral outgrowth showing the porous inner organization.

Ossicles were isolated from *Schizocardium californicum* of the family Spengelidae. Seven ossicles were found in approximately 5 mm long sections of the post-hepatic trunk. These ossicles were formed of one or more irregular smooth to rugose lobes (figure 6). The ossicles had polyhedral outgrowths at the ends (6*a,b,d*). The surface texture had a coarse appearance with pores (diameter less than 0.1 µm; figure 6*c*). The inside of the ossicle was porous (diameter less than 0.3 µm; figure 6*d*). These ossicles were the largest observed in this study, with a mean length of 26.0 ± 4.6 µm (*n* = 7) and a maximum length of 31.3 µm.

**Figure 7.**
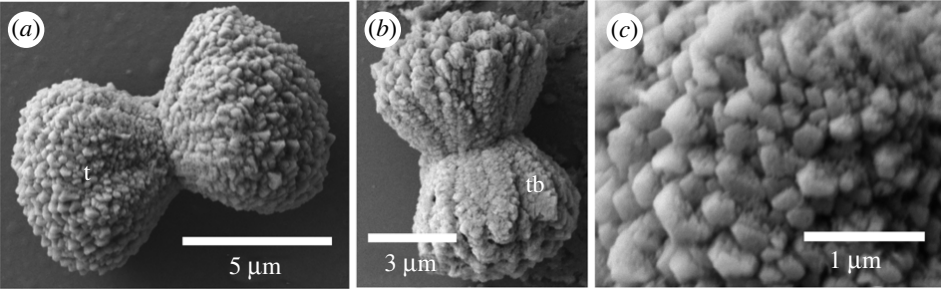
Scanning electron micrographs of ossicles from *Balanoglossus occidentalis*. (*a*) Sideview of a typical ossicle formed of two conical terminal lobes (t) joined at a point. (*b*) A smaller ossicle showing clear trabeculae (tb). (*c*) Close-up on the terminal lobe of (*a*) showing the aggregation of polyhedral crystals.

Ossicles were isolated from three species of the family Ptychoderidae and showed the full range of ossicle morphology. Those of *Balanoglossus occidentalis* were hourglass or hyperboloid shaped with convex terminal lobes (figure 7). They had mean length of 9.2 ± 1.2 µm (*n* = 12). The ossicles were uncommon. Twelve were isolated and recovered from the hepatic and dark post-hepatic trunk sections of three worms. The shape was of two opposing cones that were wide distally and narrow at the mid-region. The ossicles lacked a central shaft. Trabeculae were difficult to define in the largest ossicles of about 10 µm (figure 7*a*) but were apparent in the smallest (around 7 µm), where they formed rugose columns, longer than wide, arranged in a bouquet (figure 7*b*). A trabecula was composed of an aggregate of small polyhedral crystals (figure 7*c*). Ossicle without trabeculae was also formed of an aggregation of polyhedral crystals (figure 7*a*).

**Figure 8.**
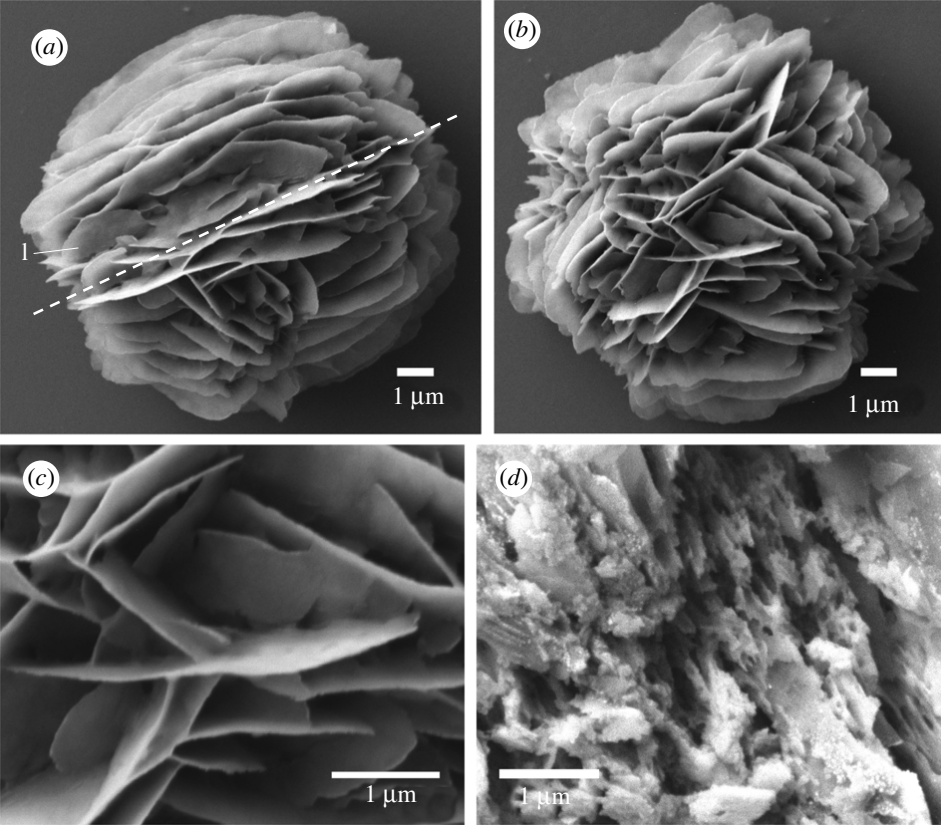
Scanning electron micrographs of ossicles from *Balanoglossus aurantiacus.* (*a*) A typical ossicle with an axis (dashed line) around which the lamellae (l) are organized. (*b*) An ossicle without such axis. (*c*) Close-up on the lamellae showing how they intersect in a network fashion. (*d*) Close-up on a broken ossicle showing the inner network of pores.

A single specimen of *Balanoglossus aurantiacus* was digested and over 50 ossicles were isolated from the trunk section posterior to the hepathic sacs. These ossicles were shaped akin to desert rose crystals. They were spherical or ellipsoid-shaped clusters of lamellae with a mean length of 13.0 ± 1.6 µm (*n* = 20) (figure 8). The lamellae were arranged around a common axis in 80% of the ossicles, otherwise they were arranged haphazardly (figure 8*a,b*). The lamellae surfaces were smooth (figure 8*c*). The core of the ossicle was formed by a network of pores (figure 8*d*).

**Figure 9.**
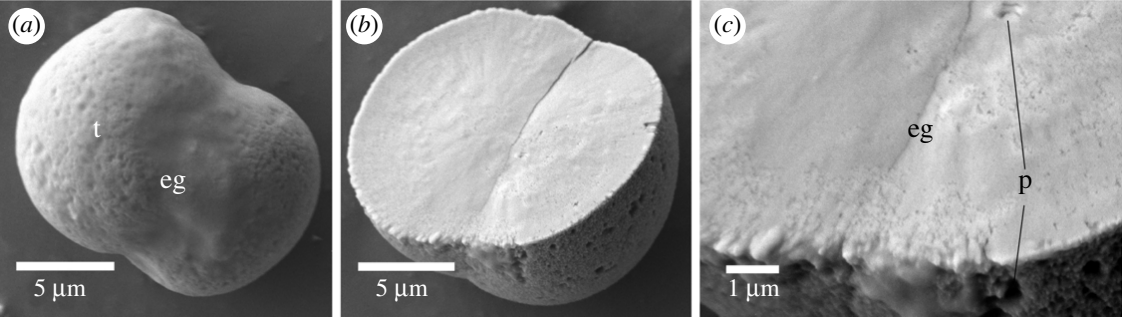
Scanning electron micrographs of ossicles from *Glossobalanus berkeleyi*. (*a*) A typical ossicle formed of two spherical terminal lobes (t) separated by an equatorial groove (eg). (*b*) A broken ossicle with a solid interior. (*c*) Close-up of the edge of the broken ossicle. Minute pores (p) can be seen inside the ossicle. Scale bar: (*a*) 5 µm, (*b*) 5 µm and (*c*) 1 µm.

A single specimen of *Glossobalanus berkeleyi* was digested and about 50 ossicles were found from the post-hepatic trunk. These ossicles were composed of a pair of conjoined, solid, spheres with a mean length of 18.1 ± 3.3 µm (*n* = 20) that had a smooth to rugose surface (figure 9). The surfaces had minute pores and canals (from less than 0.1 µm to 0.3 µm; figure 9).

### 3.2. Ossicle biomineral composition

**Figure 10.**
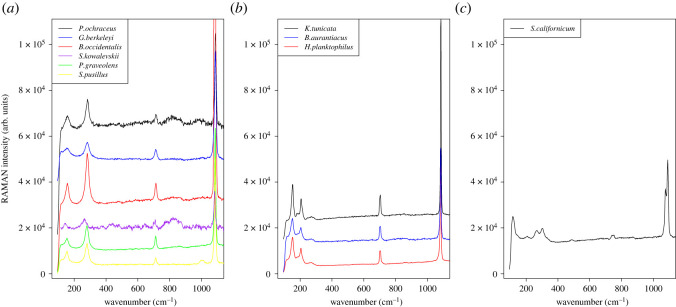
Raman spectra of enteropneusts ossicles. The peak pattern is characteristic of each polymorph. (*a*) The spectra of *G. berkeleyi, B. occidentalis, S. kowalevskii, P. graveolens* and *S. pusillus* all show the same peak pattern as the calcite ossicle from the seastar *Pisaster ochraceus.* (*b*) The ossicles of *B. aurantiacus* and *H. planktophilus* show the same peaks as the aragonite shell of the chitin *Katharina tunicata*. (*c*) The ossicles of *S. californicum* has peaks that correspond to vaterite (fig. 3 from [8]). The background noise was removed by subtracting a simple polynomial function to the spectra which does not alter the positioning or relative amplitude of the peaks. An arbitrary value was also added to each spectrum to prevent them from overlapping when plotted.

**Figure 11.**
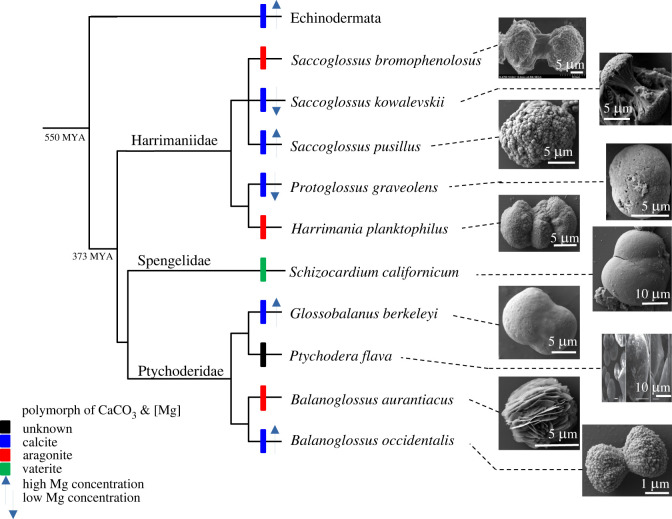
Phylogenetic tree of biomineralization in Ambulacraria*.* The phylogenetic tree is based on [20]*.* The CaCO_3_ mineral polymorph of each species is represented by a coloured tree branch. Parsimony shows calcite as the ancestral state (L = 5).

To confirm that acorn worm ossicles were composed of calcium carbonate, and to determine what polymorphs of calcium carbonate were present, Raman spectra were collected from at least two ossicles from each of the acorn worm species and compared to those of the control samples and to figure 1 of [29]. All species displayed a single polymorph. The controls were a calcite ossicle from the echinoderm *Pisaster ochraceus* and an aragonite plate from the mollusc *Katarina tunicata*. Ossicles of *S. kowalevskii*, *S. pusillus, P. graveolens, G. berkeleyi* and *B. occidentalis* were calcite. Those of *H. planktophilus* and *B. aurantiacus* were aragonite, and those of the spengelid *S. californicum* were vaterite (figures 10 and 11), demonstrating that all three calcium carbonate polymorphs are present in acorn worms. We also measured magnesium concentration in the acorn worm ossicles because echinoderm ossicles are classified as high-magnesium carbonate (greater than 4% mol MgCO_3_) and sea stars generally have the highest concentrations [30]. The calculation of magnesium concentration confirmed high-Mg calcite for the sea star *Pisaster ochraceus* and the acorn worms *S. pusillus, G. berkeleyi* and *B. occidentalis* (table 1). The highest concentrations were from *P. ochraceus* (11.4% mol) and *G. berkelyi* (12.4% mol). Low-Mg calcite was found for *S. kowalevskii* and *P. graveolens* (table 1).

**Table 1.** Calculated magnesium concentration in calcite samples. *L*, *ν*4 and *ν*1 are three major peaks of the calcite spectra. The lateral shift of those peaks is associated with magnesium concentration. The equations used to calculate the magnesium concentration are: *L* = 280.7 + 0.29 [% mol MgCO_3_], *ν*4 = 711.9 + 0.19 [% mol MgCO_3_] and *ν*1 = 1086.1 + 0.18 [% mol MgCO_3_].

vibrational mode	*Pisaster ochraceus*	*Saccoglossus kowalevskii*	*Saccoglossus pusillus*	*Protoglossus graveolens*	*Glossobalanus berkeleyi*	*Balanoglossus occidentalis*
*L* (cm^−1^)	284	263	282	280.1	284.3	282
(mol%)	11.37	<0.1	4.48	<0.1	12.4	4.48
*ν*_4_ (cm^−1^)	717	709.5	711.3	711.3	711.5	713
(mol%)	26.8	<0.1	<0.1	<0.1	−2.11	5.79
*ν*_1_ (cm^−1^)	1088	1082	1084	1086.4	1086.4	1082
(mol%)	10.5	<0.1	<0.1	1.67	1.67	<0.1
average (mol%)	16.22	<0.1	<0.1	<0.1	4	<0.1

## 4. Discussion

Here we describe the shape and mineral composition of ossicles from eight species of acorn worm, adding to the two already known [15], and demonstrate that ossicles occur in all three major families. The calcium carbonate ossicles of acorn worms are calcite, aragonite or vaterite. The first two polymorphs are widespread whereas vaterite was unique to the spengelid *Schizocardium californicum*. Mapped onto a phylogenetic tree for the Ambulacraria (figure 11), calcite is the most parsimonious ancestral polymorph, replaced by aragonite three times and vaterite one time in the Enteropneusta clade. This, coupled with the finding that three of the five acorn worm species tested had a high concentration of magnesium (figure 11) is significant because echinoderm ossicles are made of high magnesium calcite [30].

The mean lengths of acorn worm ossicles range from 3 to 30 µm, with most between 10 and 20 µm, and the largest found in *Schizocardium californicum* (30.8 µm) and *Saccoglossus bromophenolosus* (30.0 µm) [15]. A double-ended broccoli shape was conserved among species of *Saccoglossus*. *Saccoglossus kowalevskii* bilobed ossicles were similar to those of *S. bromophenolossus* but the shaft was columnar with a square cross-section (figure 2*a*) whereas those of *S. bromophenolossus* are composed of laminar layers, and circular in cross-section [15]. The trabeculae that form the terminal lobes of *S. kowalevskii* were columnar with a square cross-section and pointed tip (figure 2*b,c*), whereas those of *S. bromophenolosus* are composed of stacked, laminar crystals [15]. *Saccoglossus kowalevskii* and *S. pusillus* had a second prismatic-shaped ossicle. We suspect that these may be an early developmental stage of a double broccoli-shaped ossicle, which have a prism-shaped shaft (figure 2*d*). Also, the prismatic-shaped ossicles of *S. kowalevskii* have a shorter mean length than ossicles with lobes. Finally, the lobe widths of double-ended broccoli-shaped ossicles increase with the total length of these ossicles (electronic supplementary material, figure S1).

Ossicle shape was variable within the ptychoderids and between the two *Balanoglossus* species. A globular shape characterized the ossicles of *Glossobalanus berkeleyi* (figure 9) and *Ptychodera flava*. Unique desert rose-shaped ossicles were found in *B. aurantiacus* (figure 8), whereas the ossicles of *B. occidentalis* (figure 7) were a double-ended broccoli shape, like those of saccoglossids, though the trabeculae had a different ultrastructure. The trabeculae were an aggregation of polyhedral crystals in *B. occidentalis* whereas they are individual columnar crystals in *S. kowalevskii,* or stacks of lamellar crystals in *S. bromophenolosus*. This shape variability within the family, and between the two *Balanoglossus* species suggest that the development of ossicles in ptychoderids is unconstrained, and perhaps without function. This morphological variation may hint that a wider sampling of acorn worm ossicles could reveal more forms. Variability then, may be the rule, and the uniform shape observed in *Saccoglossus* may be unusual.

Globular-shaped ossicles that had a smooth to rugose texture were found in all three families including the harrimaniid *Protoglossus graveolens* (figure 4), the spengelid *Schizocardium californicum* (figure 6)*,* and the ptychoderids *Glossobalanus berkeleyi* (figure 9) and *Ptychodera flava* [15]. Ossicles from *P. graveolens* and *S. californicum* were both globose with a rough texture. Those of *P. graveolens* and *G. berkeleyi* show some evidence of two lobes, suggestive of the distinct bilobed ossicles of *Saccoglossus*. *Schizocardium californicum* ossicles are unique with their polyhedral outgrowth, and both ptychoderids are more alike, although ossicles from *G. berkeleyi* are bilobed with a solid core, whereas those of *P. flava* are spherical and composed of an outer layer around a porous interior [15]. Transmission electron micrographs of sections through the epithelium of *S. bromophenolosus* showed that early stage broccoli-shaped ossicles begin development as amorphous liquid entrapped inside of an extracellular sheath formed by sclerocyte cells [15]. Simple globular, or spherical ossicles may be the default shape; a shape that lacks genetic influence. It may also be plesiomorphic to Enteropneusta. If this is true, then the double-broccoli shaped ossicles with a porous texture that is reminiscent of echinoderm stereom [15] evolved independently from echinoderm stereom.

### 4.1. The origin of ambulacrarian ossicles and the echinoderm skeleton

Our data suggest that the ancestor to the Ambulacraria was a worm that possessed microscopic, monotypic, globular shaped, calcite ossicles, with low to high Mg content. These ossicles formed in an extracellular occluded space lined by the sheath of sclerocyte cells [15]. This ancestor would have had MSP130, matrix metalloproteases and carbonic anhydrases proteins [15]. These ossicles lacked intercalation with other ossicles [15,31] and were acquired in the Ediacaran. From those microscopic precursors, the echinoderms would later evolve the complex, polymorphic, intercalated, ossicles with stereom in early Cambrian calcite seas [32]. By 510 million years ago echinoderms had diversified rapidly into four distinct body plans [33–35]. The skeleton probably evolved 10–15 million years earlier [33], and the ossicles 559 million years ago [36] in the worm ancestor to hemichordates and echinoderms. A plausible explanation as to why only high concentrations of magnesium are found on the echinoderm line may relate to the structural demands of a plated endoskeleton. Ossicles were lost on the pterobranch line. On the Enteropneusta line the ossicles are calcite, vaterite or aragonite, microscopic and variable in microstructure. This suggests that there is little to no selective pressure on the CaCO_3_ polymorph or for large ossicle size in acorn worms. Their small size would, in turn, explain why they have not been detected in the fossil record. The function of acorn worm ossicles is unknown but given their location in the surface epidermis and minute size, they might serve in predator deterrence, provide grip on the walls of a burrow or tube, as storage of metabolic waste, or as calcium stores to regulate blood pH, rather than as an endoskeleton function seen in fossil and crown group Echinodermata.

## Data Availability

The data file with ossicle size measurement used to get the mean sizes and electronic supplementary material, figure S1 as well as the raw Raman data and R code used to determine the polymorph of enteropneusts ossicle are accessible at: https://doi.org/10.5281/zenodo.5103051 [37]. The data are provided in electronic supplementary material [38].
